# Correlation of Dyslipidemia and Inflammation With Obstructive Sleep Apnea Severity

**DOI:** 10.3389/fphar.2022.897279

**Published:** 2022-05-25

**Authors:** Viseslav Popadic, Milica Brajkovic, Slobodan Klasnja, Natasa Milic, Nina Rajovic, Danica Popovic Lisulov, Anica Divac, Tatjana Ivankovic, Andrea Manojlovic, Novica Nikolic, Lidija Memon, Marija Brankovic, Maja Popovic, Ana Sekulic, Jelica Bjekic Macut, Olivera Markovic, Sinisa Djurasevic, Maja Stojkovic, Zoran Todorovic, Marija Zdravkovic

**Affiliations:** ^1^ University Clinical Hospital Center Bezanijska kosa, Belgrade, Serbia; ^2^ Institute for Medical Statistics and Informatics, Faculty of Medicine University of Belgrade, Belgrade, Serbia; ^3^ Department of Internal Medicine, Division of Nephrology and Hypertension, Mayo Clinic, Rochester, MI, United States; ^4^ Faculty of Medicine, University of Belgrade, Belgrade, Serbia; ^5^ Faculty of Biology, University of Belgrade, Belgrade, Serbia; ^6^ School of Medicine, University of Belgrade, Belgrade, Serbia

**Keywords:** obstructive sleep apnea, Dyslipidemia, Inflammation, polysomnography, hypoxia

## Abstract

**Introduction:** Obstructive sleep apnea (OSA) is a serious condition linked with various metabolic disorders and associated with increased all-cause and cardiovascular mortality. Although the potential mechanisms of pathophysiological processes related to OSA are relatively well known, the data regarding the correlation between obstructive sleep apnea, dyslipidemia, and systemic inflammation are still inconclusive.

**Methods:** The study was conducted as a retrospective cohort study including 328 patients with newly diagnosed obstructive sleep apnea during the period between April 2018, and May 2020, in University Clinical Hospital Center “Bezanijska kosa”, Belgrade, Serbia. Polysomnography was performed in all patients according to the protocol. Numerous demographic, antropometric, laboratory, and clinical data were correlated to Apnea-Hypopnea Index (AHI) as a dependent variable, with a particular review on the relation between lipid abnormalities, inflammatory parameters, and obstructive sleep apnea severity. Multivariate logistic regression model was used to assess predictors of severe OSA (AHI ≥30 per hour).

**Results:** A total of 328 patients were included in the study. The mean age of the patients was 54.0 ± 12.5 years and more than two-thirds were male (68.8%). The majority of the patients had an AHI of at least 30 events per hour. Patients with severe OSA were more frequently male, obese, hypertensive and hyperlipidemic, and had increased neck circumference (both male and female patients). One hundred and thirty-two patients had metabolic syndrome. Patients with severe OSA more frequently had metabolic syndrome and significantly higher levels of glucose, creatinine, uric acid, AST, ALT, CK, microalbumine/creatinine ratio, triglyceride, total cholesterol, HDL, total cholеsterol to HDL‐C ratio, CRP, and ESR. In the multivariate linear regression model with AHI (≥30 per hour) as a dependent variable, of demographic and clinical data, triglycerides ≥1.7 mmol/L and CRP >5 mg/L were significantly associated with AHI≥30 per hour.

**Conclusion:** The present study on 328 patients with newly diagnosed obstructive sleep apnea revealed significant relation of lipid abnormalities, inflammatory markers, and other clinically important data with obstructive sleep apnea severity. These results can lead to a better understanding of the underlying pathophysiological processes and open the door to a new world of potentially useful therapeutic modalities.

## Introduction

Obstructive sleep apnea (OSA) is characterized by repetitive upper airway obstructions resulting in intermittent hypoxia and sleep fragmentation caused by arousals (Sforza and Roche, 2016). Approximately 13% of men and 6% of women have moderate to severe OSA, more often in the elderly and individuals with increased body mass index (Morsy et al., 2019). The most common symptoms include daytime sleepiness, loud snoring, and restless sleep. OSA is often underdiagnosed and unrecognized in clinical settings and is estimated that up to 26% of adults between the ages of 30 and 70^ ^years have it (Peppard et al., 2013). The severity of obstructive sleep apnea is estimated through the number of apneas or hypopneas and the number of episodes of oxygen desaturation recorded per hour of sleep during the polysomnographic study (Semelka et al., 2016).

Obstructive sleep apnea is linked with various significant risk factors and conditions that can seriously affect further prognosis and treatment including hypertension, atherosclerosis, metabolic syndrome, inflammation, insulin resistance, and diabetes melitus (Bonsignore et al., 2019). Intermittent hypoxia in patients with obstructive sleep apnea leads to increased oxidative stress and free radicals production which has been suggested as a potential mechanism of a plethora of pathophysiological processes such as endothelial dysfunction, increased sympathetic activity, systemic inflammatory response, impaired glucose and lipid metabolism (Arnaud et al., 2009).

Previously published paperworks showed a strong correlation between obstructive sleep apnea and dyslipidemia (Gündüz et al., 2018; Gündüz et al., 2019). Excessive increase in total cholesterol or triacylglycerols carries increased cardiovascular risk, mainly due to the acceleration of the atherosclerotic process. However, it is shown that abnormal lipid clearance is probably responsible for this mechanism due to decreased activity of lipoprotein lipase and subsequent formation of LDL subclass B, which oxidation leads to progressive vascular damage (Mirrakhimov and Ali, 2013). The activity of lipoprotein lipase is maintained by insulin and decreased by cortisol and epinephrine. This is why various studies emphasized the role of insulin resistance and increased sympathetic activity as the most important pathological mechanisms related to obstructive sleep apnea (Jung et al., 2016; Almendros and García-Río, 2017). Also, different animal models showed that not just the presence, but the severity of intermittent hypoxia is strongly related to hyperlipidemia and liver oxidative stress, resulting in an increased lipolysis, decreased lipoprotein clearance, and enhanced lipid output (Drager et al., 2010).

Systemic inflammation is an important aspect in patients with OSA. Intermittent hypoxia induces activation of inflammatory cells, the release of inflammatory mediators, and consequent vascular pathophysiology. A large number of studies demonstrated increased levels of inflammatory biomarkers and their decrease with forehand and proper therapy (Orrù et al., 2020). It is shown that certain inflammatory parameters including CRP, fibrinogen, IL-6, and Tumor Necrosis Factor-α (TNF-α) are linked with higher cardiovascular risk in patients with obstructive sleep apnea (Ryan et al., 2009). Besides the fact that these markers correlate with the extent of hypoxia during sleep, the novel studies suggest the theory of OSA-specific factors that predispose to inflammation and the subset of individuals susceptible to inflammation (Unnikrishnan et al., 2015).

Although the potential mechanisms of various conditions linked with OSA are relatively well known, the data regarding the correlation between obstructive sleep apnea, dyslipidemia, and systemic inflammation are still inconclusive.

Our study aimed to correlate lipid abnormalities and inflammatory markers with the severity of obstructive sleep apnea in newly diagnosed patients with OSA.

## Materials and Methods

The study was conducted as a retrospective cohort study including 328 patients with newly diagnosed obstructive sleep apnea during the period between April 2018, and May 2020, in University Clinical Hospital Center “Bezanijska kosa”, Belgrade, Serbia, at the Department of Pulmonology, The Section for diagnostics and treatment of obstructive sleep apnea. All participants over 18^ ^years of age who had been referred for polysomnography (PSG) because of suspected sleep-related breathing disorders and with at least 3.5 h of sleep were included in the study. The exclusion criteria were the presence of contact allergic reactions to electrode, the presence of infection and surgical wound on the skin in contact with electrode, the presence of sleep-related movement disorders, unstable vital signs, major behavioral or neurological disorders, the use of medications that could affect sleep or autonomic nervous system function. All participants provided written informed consent.

### Polysomnography (PSG)

To establish the diagnosis of obstructive sleep apnea and estimate its severity, the polysomnographic study was performed with the Alice four Sleep System (Respironics Inc., Murrysville, PA, United States). Sleep parameters were evaluated in accordance with the 2007 American Academy of Sleep Medicine (AASM) protocol (Berry et al., 2012). Apnea-Hypopnea Index (AHI - the number of apneas or hypopneas recorded during the study per hour of sleep) was derived from level 1 polysomnography (PSG). Apneas were defined as cessations of nasal flow lasting 10 or more seconds and hypopneas as a decrease of 50% or more in nasal flow and associated with 3% or more oxygen desaturation. The patients with AHI between 5 and 14 were considered to have mild sleep apnea, with AHI between 15 and 29 were considered to have moderate sleep apnea, while the patients with AHI more than 30 were considered to have severe sleep apnea. Oxygen Desaturation Index (ODI), as an index of nocturnal hypoxemia, was derived from the nocturnal pulse oximeter (NPO). The ODI was defined as the number of episodes of oxygen desaturation per hour of sleep, with oxygen desaturation defined as a decrease in blood oxygen saturation (SpO2) to lower than 3% below baseline. The baseline value was determined as the average SpO2 during the first 3 min of recording. The pulse oximeter was worn on the index finger of the non-dominant hand. Epworth sleepiness scale was used to measure the level of daytime sleepiness.

### Demographic, Antropometric Data, Laboratory and Clinically Significant Parameters

Demographic and anthropometric data (age, gender, BMI, neck, waist, and hip circumference), past medical history (hypertension, diabetes mellitus, hyperlipidemia, coronary heart disease), and data regarding smoking, alcohol abuse, and physical activity were collected. Laboratory parameters including urea, glycemia, bilirubin, alanine (ALT) and aspartate transaminase (AST), lactate dehydrogenase (LDH), creatine kinase (CK), gamma-glutamyltransferase (GGT), potassium, sodium, magnesium, chloride, amylase, creatinine, uric acid, serum albumins, serum proteins, C-reactive protein, triglycerides, total cholesterol, low-density lipoprotein (LDL), high-density lipoprotein (HDL), non-high-density lipoprotein (non-HDL), complete blood count, erythrocyte sedimentation rate (ESR), microalbuminuria in a free urine sample, thyroid-stimulating hormone (TSH), free thyroxine (FT4), Estimated Glomerular Filtration Rate (eGFR), high sensitive troponin T (hsTnT), N-Terminal pro-Brain natriurethic peptide (NT-proBNP), glycated hemoglobin (HbA1c), and fibrinogen were followed. Participants were also followed in terms of metabolic syndrome presence and its correlation with OSA severity. Metabolic syndrome was defined according to the definition of National Cholesterol Education Program (NCEP-R), NCEP Adult Treatment Panel (ATP)-III (Expert Panel on Detection, Evaluation, and Treatment of High Blood Cholesterol in Adults, 2001). The data regarding lipid abnormalities and inflammation were correlated with the severity of obstructive sleep apnea (expressed as AHI value) and other laboratory and clinically significant parameters. Reference values for laboratory, clinical, and other significant parameters are provided in Supplementary Table S1.

### Statistical Analysis

Numerical data were presented as mean with 95% confidence interval, or median with minimum and maximum value. Categorical variables were summarized by absolute numbers with percentages. Differences in demographic, clinical, lipids and inflammation parameters according to OSA severity were assessed by ANOVA with LSD for post hoc analyses or Kruskal Wallis test with Mann Whitney test for post hoc analyses (for numerical data according to data distribution) or by Chi square test for categorical variables. Multivariate logistic regression model was used to assess predictors of severe OSA (AHI ≥30 per h). Characteristics of the patients with OSA were first assessed by univariate logistic regression analysis, following with the final model being developed using a forward stepwise (wald) multivariate logistic regression analysis. The characteristics pool for stepwise-regression modeling was defined based on characteristics correlation with AHI≥30 per hour or known relevance. The VIF (variance inflation factor) was used to examine colinearity between covariates. The goodness of fit was evaluated by Hosmer and Lemeshow Test (Chi square = 0.216, df = 2, *p* = 0.897). In all analyses, the significance level was set at 0.05. Statistical analysis was performed using IBM SPSS statistical software (SPSS for Windows, release 25.0, SPSS, Chicago, IL).

### Ethics

The study was organized according to the principles of the Declaration of Helsinki of 1975, as revised in 2008 and approved by the Ethics Committee of University Clinical Hospital Center “Bezanijska kosa”.

## Results

A total of 328 patients were included in the study. Ninety-seven patients (29.6%) had an AHI less than fifteen events per hour, 25.3% had an AHI of at least 15 events per hour, but fewer than 30, and 45.1% of patients had an AHI of at least 30 events per hour. The mean age of the patients was 54.0 ± 12.5 years and more than two-thirds were male (68.8%). Patients with AHI of at least 30 events per hour were more frequently male, obese, hypertensive and hyperlipidemic, and had increased neck circumference (both male and female patients) (Table 1). Detailed demographic data and comorbidities of the study population according to OSA severity are presented in Table 1.

**TABLE 1:** Demographic data and comorbidities of the study population according to OSA severity.

Variable	AHI		
	I ≤ 15 per h (*n* = 97)	II ≥ 15, but <30 per h (*n* = 83)	III ≥30 per h (*n* = 148)
Age, mean (95%CI), years	51.9 (49.1–54.6)	56.0 (53.5–58.6)	54.4 (52.4–56.4)
Male gender, n (%)	53 (54.6)	52 (62.7)	120 (81.1)^a^ ^,^ ^b^
Smoking, yes, n (%)	27 (31.8)	24 (30.4)	46 (33.1)
Neck circumference, mean (95%CI), cm			
Male	42.1 (41.1–43.2)	43.5 (42.8–44.2)	44.1 (43.5–44.7)^a^ ^,^ ^b^
Female	38.7 (37.4–40.1)	39.3 (38.5–40.1)	40.4 (39.3–41.4)^a^
BMI>30.0, n (%)	35 (36.8)	42 (50.6)	107 (73.3)^a^ ^,^ ^b^
Hypertension, n (%)	38 (39.2)	50 (60.2)^c^	95 (64.2)^a^
Hyperlipidemia, n (%)	26 (26.8)	39 (47.0)^c^	77 (52.0)^a^
Diabetes mellitus, n (%)	13 (13.4)	18 (21.7)	34 (23.0)
Myocardial infarction, n (%)	2 (2.8)	4 (12.1)	8 (12.7)
Patients on lipid lowering therapy, n (%)	6 (8.5)	5 (15.2)	11 (17.5)

a III, vs. I < 0.05.

b III, vs. II < 0.05.

c II, vs. I < 0.05.

Clinical, lipids and inflammation parameters of the study population according to OSA severity are presented in Table 2. Patients with AHI of at least 30 events per hour had significantly higher levels of glucose, creatinine, uric acid, AST, ALT, CK, microalbumine/creatinine ratio, triglyceride, lower levels of HDL, total cholesterol to HDL-C ratio, CRP, and ESR (Table 2).

**TABLE 2:** Clinical, lipids and inflammation parameters of the study population according to OSA severity.

Variable	AHI		
	I ≤ 15 per h (*n* = 97)	II ≥ 15, but <30 per h (*n* = 83)	III ≥30 per h (*n* = 148)
Glucose (mmol/L)	5.7 (5.4–6.0)	6.1 (5.705–6.530)	6.3 (5.991–6.684)^a^
Creatinine (umol/L)	81.3 (77.6–85.0)	84.2 (80.4–88.0)	90.8 (87.2–94.4)^a^ ^,^ ^b^
Uric acid (umol/L)	337.6 (316.2–359.0)	327.6 (307.8–347.4)^c^	375.7 (359.4–391.9)^a^ ^,^ ^b^
Albumin (g/L)	45.1 (44.1–46.1)	43.3 (42.1–44.5)	44.8 (44.1–45.5)
Direct bilirubin (U/L)	2.4 (2.2–2.7)	2.6 (2.2–3.0)	2.4 (2.2–2.7)
Total bilirubin (U/L)	12.6 (11.4–13.8)	13.3 (11.3–15.2)	12.5 (11.2–13.7)
AST (U/L)	21.6 (20.1–23.2)	21.7 (19.2–24.1)	25.3 (21.9–28.7)^a^
ALT (U/L)	24.8 (21.3–28.2)	22.8 (18.1–27.4)	31.6 (26.1–37.2)^a^ ^,^ ^b^
CK (U/L)	96.7 (84.4–109.0)	102.8 (85.0–120.6)	135.8 (94.6–177.0)^a^
Gama GT (U/L)	32.2 (24.3–40.1)	27.5 (22.0–33.0)	36.7 (30.9–42.6)
Microalbumine/Creatinine Ratio	1.8 (0.15–3.5)	1.9 (1.1–2.7)	10.1 (2.6–17.6)^a^ ^,^ ^b^
Triglyceride (mmol/L)	1.8 (1.6–2.0)	1.9 (1.7–2.1)^c^	2.3 (2.1–2.5)^a^ ^,^ ^b^
Total cholesterol (mmol/L)	4.7 (4.3–5.0)	3.4 (3.0–3.9)	3.7 (3.3–4.0)^a^
HDL (mmol/L)	1.3 (1.2–1.4)	1.3 (1.2–1.4)	1.2 (1.1–1.2)^a^
LDL (mmol/L)	3.6 (3.4–3.8)	3.6 (3.4–3.8)	3.6 (3.4–3.8)
Non-HDL (mmol/L)	4.3 (4.1–4.5)	4.3 (3.9–4.7)	4.3 (4.0–4.6)
Total chol./HDL-C	4.4 (4.2–4.8)	4.7 (4.3–5.1)	4.9 (4.6–5.2)^a^
LDL-C/HDL-C	2.8 (2.6–3.0)	2.9 (2.5–3.2)	2.9 (2.7–3.2)
CRP^*^ (mg/L)	2.3 (0.9–5.7)	4.0 (1.4–6.0)	4.5 (2.0–8.0)^a^ ^,^ ^b^
ESR^*^ (mm/h)	14.0 (7.0–20.0)	13.5 (10.0–25.0)	17.0 (10.5–26.5)^a^

Data are presented as mean (95%CI).

* Median (25–75^th^ Percentile).

a III, vs. I < 0.05.

b III, vs. II < 0.05.

c II, vs. I < 0.05.

One hundred and thirty-two patients had metabolic syndrome. Patients with metabolic syndrome had significantly more present prior myocardial infarction than patients without metabolic syndrome (*p* = 0.048). Male patients with metabolic syndrome had increased neck circumference (*p* < 0.001) (Table 3).

**TABLE 3:** Demographic data and comorbidities of the study population according to metabolic syndrome.

Variable	Metabolic syndrome	
	No (*n* = 198)	Yes (*n* = 132)
Age, mean ± sd, years	53.0 (51.1–54.9)	55.5 (53.6–57.4)
Male gender, n (%)	139 (70.2)	88 (66.7)
Neck circumference, mean (95%CI), cm		
Male	43.0 (42.5–43.5)	44.6 (43.9–45.3)^a^
Female	39.0 (38.2–39.7)	39.9 (38.8–41.0)
Smoking, yes, n (%)	59 (33.3)	40 (31.3)
Myocardial infarction, n (%)	4 (4.4)	10 (12.8)^a^

a p < 0.05.

Clinical, lipids and inflammation parameters of the study population according to metabolic syndrome are presented in Table 4. Patients with metabolic syndrome had significantly higher values of uric acid, gama GT, microalbumine/creatinine ratio, CRP, and ESR than patients without metabolic syndrome (*p* < 0.05) (Table 4).

**TABLE 4:** Clinical, lipids and inflammation parameters of the study population according to metabolic syndrome.

Variable	Metabolic syndrome	
	No (*n* = 198)	Yes (*n* = 132)
Creatinine (umol/L)	86.6 (84.4–88.9)	85.9 (81.5–90.2)
Uric acid (umol/L)	328.3 (315.3–341.4)	388.2 (370.4–406.0)^a^
Albumin (g/L)	44.7 (43.9–45.4)	44.6 (44.8–45.4)
Direct bilirubin (U/L)	2.5 (2.3–2.7)	2.4 (2.2–2.7)
Total bilirubin (U/L)	13.2 (12.1–14.2)	12.2 (11.0–13.4)
AST (U/L)	22.7 (21.0–24.4)	23.3 (20.7–25.9)
ALT (U/L)	25.1 (21.5–28.7)	29.0 (24.9–33.1)
CK (U/L)	107.6 (91.1–124.0)	119.9 (88.5–151.3)
Gama GT (U/L)	27.6 (23.4–31.8)	38.8 (31.6–46.0)^a^
Microalbumine/Creatinine	2.1 (0.75–3.4)	8.1 (2.1–14.2)^a^
CRP^*^ (mg/L)	2.7 (1.3–6.0)	4.7 (1.9–8.0)^a^
ESR^*^ (mm/h)	13.0 (6.0–20.0)	18.5 (10.5–26.0)^a^

Variables are presented as mean (95%CI).

* Median (25–75^th.^Percentile).

a p < 0.05.

**FIGURE 1:**
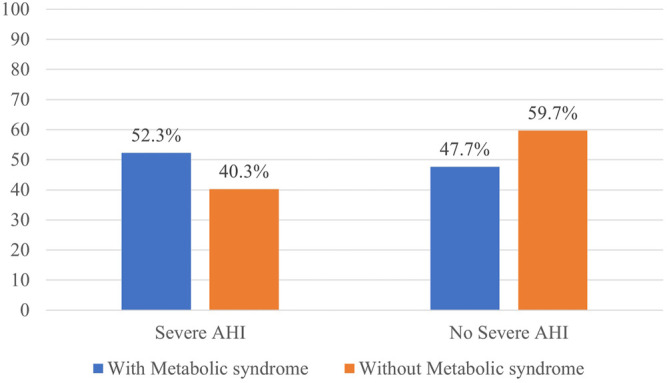
Distribution of the patients with and without metabolic syndrome according to OSA severity.

Patients with severe AHI (≥30 per hour) more frequently had metabolic syndrome (Figure 1).

In the multivariate logistic regression model with AHI (≥30 per hour) as a dependent variable, of demographic and clinical data, triglycerides ≥1.7 mmol/L and CRP >5 mg/L were significantly associated with AHI≥30 per hour (*p* = 0.003 and *p* = 0.021, respectively) (Table 5).

**TABLE 5:** Multivariate logistic regression model with AHI as an dependent variable.

Variable	p	OR	95%CI for OR
Triglycerides ≥1.7 mmol/L	0.003	1.961	1.251–3.072
CRP >5 mg/L	0.021	1.724	1.086–2.736

## Discussion

In this retrospective cohort study on 328 patients with newly diagnosed obstructive sleep apnea, we showed a significant correlation of lipid abnormalities and inflammatory markers with Apnea-Hypopnea Index (AHI) as an indicator of obstructive sleep apnea severity.

Lipid abnormalities associated with OSA severity in our study were triglycerides, high-density lipoprotein (HDL), total cholesterol, and total cholesterol to HDL ratio. These lipid abnormalities were more pronounced in patients with severe obstructive sleep apnea (AHI≥30 per h), while we observed no difference between AHI groups in terms of lipid-lowering therapy usage. *Guscoth et al.* recently showed the correlation between triglycerides and severity of obstructive sleep apnea even in patients without central adiposity (with normal waist circumference), which is extremely significant as there is an ingrained opinion that only obese patients with high BMI are susceptible to obstructive sleep apnea and its serious forms (Guscoth et al., 2021). The main mechanisms involved in higher cholesterol and triglycerides levels in patients with OSA are increased hepatic triglyceride and cholesterol production, decreased clearance by lipase activity inhibition, and increased free fatty acid mobilization from adipose tissue (Drager et al., 2018). It is noted that intermittent hypoxia initiates upregulation of important transcription factors in the process of cholesterol and triglyceride biosynthesis (Li et al., 2005). Considering that the process of lipid production is inhibited by insulin, patients with insulin resistance showed higher lipid levels especially those with severe forms of OSA (Liu et al., 2015). In a study by *Togeiro et al.* triglycerides were marked as a strong indicator of recurrent hypoxia, independently associated with AHI (Togeiro et al., 2013).

Our study showed a significant correlation of HDL with OSA severity, indicating that patients with lower HDL values have higher Apnea-Hypopnea Index (AHI). Considering protective cardiovascular effects of high-density lipoproteins (HDL), it is shown that certain subfractions of HDL, especially small HDL subfractions including small LDL3–7 and small HDL8–10, can have atherogenic effects in patients with OSA (Kollar et al., 2021). On the other hand, bigger HDL subfractions can have impaired antioxidant activity because of increased HDL lipid peroxide levels and decreased serum paraoxonase-1 (PON1) activity, which are the two main factors related to the antioxidant activity of HDL (Yadav et al., 2014). This can be a potential explanation for a lack of evidence of protective effects of HDL in patients with OSA, even in patients with normal HDL values. It is important to note that patients with reduced antioxidant HDL activity also had increased tumor necrosis factor-α (TNF-α) and intercellular adhesion molecule 1 (ICAM-1) levels pointing out enhanced systemic inflammation in severe forms of OSA (Pak et al., 2014). A study by *Koseoglu et al.* suggests the significant role of monocyte to HDL ratio as a potential predictor of OSA severity and its relationship with increased cardiovascular risk (Inonu Koseoglu et al., 2018).

The advantages of statin therapy in the reduction of cardiovascular risk are unquestionable (Bibbins-Domingo et al., 2016; Mach et al., 2019). However, the evidence of positive effects of lipid-lowering therapy in patients with OSA is controversial, while, in general, this important subject is poorly studied. In a multicenter randomized controlled trial by *Joyeux-Faure et al.*, there was no improvement in endothelial function after 12 weeks of atorvastatin usage in patients with severe OSA (Joyeux-Faure et al., 2014). However, statin therapy improved ambulatory office blood pressure, potentially reducing the overall cardiovascular risk. The study by *Emin et al.* found that the internalization of a specific protein called CD59 in patients with OSA promoted endothelial inflammation and damage, while atorvastatin treatment stabilized CD59 protein on the endothelial cell surface, protecting them from hypoxia-induced injury and consequently increased cardiovascular risk (Emin et al., 2016). Studies estimating cardiovascular risk reduction in patients with OSA on more potent lipid-lowering drugs are missing. The beneficial effects of CPAP and statin therapy combination are still unknown and require further investigation.

Systemic inflammation, according to many authors, is one of the main pathogenetic mechanisms in patients with OSA (Vicente et al., 2016; Kheirandish-Gozal and Gozal, 2019). Intermitent hypoxia in OSA promotes a persistent low-intensity systemic inflammation consequently inducing end-organ dysfunction. *Scorza et al.* demonstrated the role of tumor necrosis factor-α (TNF-α) as a central mediator of inflammatory response in OSA (Scorza et al., 2013). Patients with obstructive sleep apnea had higher plasma, serum, and intracellular levels of TNF-α, while several studies emphasized the role of TNF-α in OSA-related cardiovascular morbidity (Ramesh et al., 2012; Cao et al., 2020). Various studies demonstrated a correlation of interleukin-6 (IL-6) and interleukin 1β (IL-1β) with OSA severity as well (Tang et al., 2017; Imani et al., 2020). CRP is a significant marker of inflammation synthesized in the liver and is dominantly under the regulation of IL-6. In our study, both CRP and erythrocyte sedimentation rate (ESR) were found to be correlated with OSA severity, while CRP was determined as a predictor of OSA severity in the multivariate linear regression model. Regarding the importance of erythrocyte sedimentation rate in patients with OSA, a study by *Lee et al.* demonstrated that ESR was more closely correlated with OSA severity than hs-CRP (Lee et al., 2016). A number of different studies, including a meta-analysis by *Nadeem et al.*, showed that a higher CRP level is associated with OSA but also with an increased cardiovascular risk as seen in patients with angina pectoris, acute coronary syndrome, and a history of myocardial infarction (Nadeem et al., 2013). The study by *Sharma et al.* found that CRP levels were higher in obese patients, associated with body mass index only, but without an independent correlation between the severity of OSA and CRP (Sharma et al., 2008). The number of participants and study group structure in terms of BMI and AHI seems to have an important impact on the results, as most of these studies had significant divergence between groups. Our study group consisted of 25.3% of patients with AHI>15 < 30 and 45.1% of patients with AHI>30, while the mean BMI was 32 kg/m^2^ (predominantly obese participants with moderate to severe OSA). Both BMI and CRP were independently associated with AHI. It is also important to note that the large number of studies determining the correlation between systemic inflammation and the severity of OSA was cross-sectional. Future studies with better methodology are needed to definitely shed light on the significance of inflammatory markers in patients with OSA.

Unregulated diabetes mellitus with advanced microvascular complications can be emphasized by the further progression of OSA probably because of the impaired neuromodulatory mechanisms and increased sympathetic activity, provoking more pronounced systemic inflammation and metabolic disorders (Shen et al., 2018). In our study group, around 20% of patients had diabetes melitus, although the results did not show a significant difference in diabetes melitus prevalence between OSA severity groups. However, glucose levels were associated with OSA severity indicating the importance of normal levels of glycemia, as obstructive sleep apnea can worsen the control of blood sugar levels and contribute to several related complications. It is important to underline that we found positive correlation between the presence of metabolic syndrome and OSA severity. In our study group, participants with severe OSA (AHI≥30 per hour) more frequently had metabolic syndrome, while those with metabolic syndrome had higher levels of CRP and ESR. This indicates the importance of low-grade systemic inflammation in developing various significant complications, including coronary artery disease, as patients with metabolic syndrome and severe OSA had significantly more present prior myocardial infarction than patients without metabolic syndrome. These results are in concordance with previously published studies (Parish et al., 2007; Castaneda et al., 2018). Other clinically important parameters, including creatinine and uric acid levels, as well as microalbumin to creatinine ratio, also showed a positive correlation with the severity of OSA. The reason for this lies in the fact that patients with progressed associated conditions including hypertension and diabetes mellitus will most likely have severe forms of OSA, but also an impaired kidney function as a result of their primary cardiovascular and metabolic conditions. This conclusion is in concurrence with numerous studies which also showed that the presence of OSA can fasten the progression of chronic kidney disease and that 50–70% of patients with end-stage renal disease have OSA (Abuyassin et al., 2015; Aziz and Chaudhary, 2016).

There are several limitations of this study. Despite being a single center study, the sample size is quite satisfactory. However, there is a considerable number of patients with previous cardiovascular and metabolic conditions, including hypertension, diabetes melitus, coronary heart disease, and hyperlipidemia. The prior usage of lipid-lowering therapy could be a possible reason for a lack of evidence regarding the relation of LDL values and OSA severity. However, we observed no difference between AHI groups in terms of lipid-lowering therapy usage. Having in mind the heterogeneity of the group, aside from various cardiovascular and metabolic conditions, it is unknown for how long these patients had OSA, as we included only newly diagnosed patients in order to estimate the relation of lipid abnormalities and inflammatory parameters with the severity of OSA, without the possible effects of continuous positive airway pressure (CPAP) therapy.

## Conclusion

Obstructive sleep apnea (OSA) is linked with several metabolic disorders and associated with increased all-cause and cardiovascular mortality. Persistent low-intensity systemic inflammation and lipid abnormalities due to intermitent hypoxia are associated with OSA severity and contribute significantly to increased cardiovascular and end-organ damage risk. Patients with more pronounced metabolic syndrome and risk factors for severe forms of OSA, including gender and body mass index among others, should promptly be evaluated for OSA presence in order to start a forehand therapy and reduce the risk of various complications. This is why ranking individuals with newly diagnosed OSA in regard to their risk is extremely important, while future studies should focus on how to establish novel noninvasive markers with appropriate sensitivity and specificity in predicting which patients are at greater risk. This can lead to a better understanding of the underlying pathophysiological processes and open the door to a new world of potentially useful therapeutic modalities.

## Data Availability

The raw data supporting the conclusions of this article will be made available by the authors, without undue reservation.

## Abbreviations

AHI, Apnea-Hypopnea Index; BMI, body mass index; CPAP, continuous positive airway pressure; CRP, C-reactive protein; ESR, erythrocyte sedimentation rate; FEV1, forced expiratory volume; FVC, forced vital capacity; HDL, high density lipoproteins; ICAM-1, intercellular adhesion molecule-1; IL-1β, interleukin-1β; IL-6, interleukin-6; LDL, low density lipoproteins; ODI, Oxygen Desaturation Index; OSA, obstructive sleep apnea; PON1, paraoxonase-1; PSG, polysomnography; TNF-α, Tumor Necrosis Factor- α.
