# Supplementary material for: Correlation of Dyslipidemia and Inflammation With Obstructive Sleep Apnea Severity
Source: Front Pharmacol. 2022 May 25;13:897279. doi: 10.3389/fphar.2022.897279 (PMC9179947; doi:10.3389/fphar.2022.897279)
Supplement: Supplementary file 1 [file Table1.DOCX]

**Supplemental Table 1.**

Reference values and scores of evaluated indices, laboratory and other clinically significant parameters

| **Body mass index (BMI, kg/m^2^)** | |
| --- | --- |
| Underweight | <18.5 |
| Normal range | 18.5-24.9 |
| Overweight | 25-29.9 |
| Obese | ≥30.0 |
| Class I | 30.0-34.9 |
| Class II | 35.0-39.9 |
| Class III | ≥40.0 |
| **Apnea-Hypopnea Index (AHI) scoring** | |
| <5 | Normal (no sleep apnea) |
| ≥5-14 | Mild sleep apnea |
| 15-29 | Moderate sleep apnea |
| ≥30 | Severe sleep apnea |
| **Oxygen Desaturation Index (ODI) scoring** | |
| <5 | Normal (no sleep apnea) |
| ≥5-14 | Mild sleep apnea |
| 15-29 | Moderate sleep apnea |
| ≥30 | Severe sleep apnea |
| **Epworth Sleepiness Scale** | |
| 0-10 | Normal range |
| 11-14 | Mild sleepiness |
| 15-17 | Moderate sleepiness |
| 18-24 | Severe sleepiness |
| **Reference values of laboratory parameters** | |
| **Biochemical parameters** | |
| Urea | 2.8-8.1 mmol/L |
| Glucose | 4.1-5.9 mmol/L |
| Total bilirubine | up to 21umol/L |
| Alanine transaminase (ALT) | up to 41 U/L in men, up to 33 U/L in women |
| Aspartate transaminase (AST) | up to 40 U/L in men, up to 32 U/L in women |
| Lactate dehydrogenase (LDH) | 270.0-450.0 U/L in men, 270.0-428.0 U/L in women |
| Creatine kinase (CK) | up to 190 U/L in men, up to 170 U/L in women |
| Gamma-glutamyltransferase (GGT) | 10.0-71.0 U/L in men, 6.0-42.0 U/L in women |
| Potassium | 3.5-5.1 mmol/L |
| Sodium | 136.0-145.0 mmol/L |
| Chloride | 98.0-107.0 mmol/L |
| Amylase | 28.0-100.0 mmol/L |
| Creatinine | 62.0-106.0 umol/L in men, 44.0-80.0 umol/L in women |
| Uric acid | 202.3-416.5 umol/L in men, 142.8-339.2 umol/L in women |
| Serum albumins | 35.0-52.0 g/L |
| Serum proteins | 64.0-83.0 g/L |
| C-reactive protein | up to 5 mg/L |
| Triglycerides | up to 1.7 mmol/L |
| Total cholesterol | up to 5.2 mmol/L |
| Low-density lipoproteins (LDL) | up to 3.4 mmol/L |
| High-density lipoproteins (HDL) | up to 1.6 mmol/L |
| Estimated Glomerular Filtration Rate (eGFR) | >90 ml/min/1.73m^2^ |
| Microalbuminuria | <30mg |
| Creatinine in free urine sample | 20.0-320.0 mg/dL in men, 20.0-275.0 mg/dL in women |
| Thyroid-stimulating hormone (TSH) | 0.55-4.78 uIU/mL |
| Free thyroxine (FT4) | 11.5-22.7 pmol/L |
| High sensitive troponin T (hsTnT) | up to 14 ng/L |
| N-Terminal pro-Brain natriurethic peptide (NT-proBNP) | up to 125 pg/mL |
| Glycated haemoglobin (HbA1c) | 4.0-6.0% |
| Fibrinogen | 2.1-4.0 g/L |
| **Complete blood count** | |
| Leukocytes | 4.0-10.0x10^9^/L |
| Erythrocytes | 4.5-6.5/10^12^/L in men, 3.8-5.8/10^12^/L in women |
| Hemoglobin | 130-170g/L in men, 115-160g/L in women |
| Hematocrit | 0.40-0.54L/L in men, 0.37-0.47L/L in women |
| Thrombocytes | 150.0-450.0x10^9^/L |
| Neutrophils | 2.0-7.5x10^9^/L |
| Lymphocytes | 1.0-4.0x10^9^/L |
| Monocytes | 0.2-1.0x10^9^/L |
| Eosinophils | up to 0.5x10^9^/L |
| Basophils | up to 0.2x10^9^/L |
| Erythrocyte sedimentation rate (ESR) | up to 12mm/h in men, up to 19mm/h in women2.1 |
